# Distinct Diet-Microbiota-Metabolism Interactions in Overweight and Obese Pregnant Women: a Metagenomics Approach

**DOI:** 10.1128/spectrum.00893-21

**Published:** 2022-03-28

**Authors:** Mrunalini Lotankar, Kati Mokkala, Noora Houttu, Ella Koivuniemi, Nikolaj Sørensen, Henrik Bjørn Nielsen, Eveliina Munukka, Leo Lahti, Kirsi Laitinen

**Affiliations:** a Institute of Biomedicine, Research Centre for Integrative Physiology and Pharmacology, University of Turku, Turku, Finland; b Clinical Microbiomics, Copenhagen, Denmark; c Medical Faculty, University of Turku, Turku, Finland; d Department of Computing, Faculty of Technology, University of Turku, Turku, Finland; e Department of Obstetrics and Gynecology, Turku University Hospital, Turku, Finland; f Department of Clinical Microbiology, Turku University Hospital, Turku, Finland; Wayne State University

**Keywords:** diet quality, metagenomic, microbiota diversity, metabolism, overweight, obesity, obese

## Abstract

Diet and gut microbiota are known to modulate metabolic health. Our aim was to apply a metagenomics approach to investigate whether the diet-gut microbiota-metabolism and inflammation relationships differ in pregnant overweight and obese women. This cross-sectional study was conducted in overweight (*n* = 234) and obese (*n* = 152) women during early pregnancy. Dietary quality was measured by a validated index of diet quality (IDQ). Gut microbiota taxonomic composition and species diversity were assessed by metagenomic profiling (Illumina HiSeq platform). Markers for glucose metabolism (glucose, insulin) and low-grade inflammation (high sensitivity C-reactive protein [hsCRP], glycoprotein acetylation [GlycA]) were analyzed from blood samples. Higher IDQ scores were positively associated with a higher gut microbiota species diversity (*r* = 0.273, *P *= 0.007) in obese women, but not in overweight women. Community composition (beta diversity) was associated with the GlycA level in the overweight women (*P *= 0.04) but not in the obese. Further analysis at the species level revealed a positive association between the abundance of species Alistipes finegoldii and the GlycA level in overweight women (logfold change = 4.74, *P *= 0.04). This study has been registered at ClinicalTrials.gov under registration no. NCT01922791 (https://clinicaltrials.gov/ct2/show/NCT01922791).

**IMPORTANCE** We observed partially distinct diet-gut microbiota-metabolism and inflammation responses in overweight and obese pregnant women. In overweight women, gut microbiota community composition and the relative abundance of A. finegoldii were associated with an inflammatory status. In obese women, a higher dietary quality was related to a higher gut microbiota diversity and a healthy inflammatory status.

## INTRODUCTION

Diet has a fundamental role in human metabolism and health. Furthermore, there is convincing evidence that various components of the diet, such as carbohydrates, fat, and fiber (1, 2), influence the composition of the gut microbiota (3). There are further factors that modulate metabolic health and the gut microbiota (i.e., the host’s nutritional status, such as overweightness and outright obesity, two conditions commonly observed today). In this respect, it has been hypothesized that the link between diet, gut microbiota and metabolic health may also be influenced by the host’s nutritional status.

The healthy human gut microbiota comprises a variety of distinct bacterial species and a reduced diversity has been associated with metabolic complications such as insulin resistance and dyslipidemia (4). In a similar manner, a good dietary quality, in accordance with national recommendations, has been demonstrated to confer health benefits in reducing the risk of type 2 diabetes and cardiovascular diseases (5, 6). Considerable work has been done to reveal this relationship, for example showing that excess adiposity of the host is linked with a range of adverse metabolic outcomes, as well as with deviations in gut microbiota composition (7, 8). The gut microbiota is malleable to changes in environment and diet (9). A recent review (10) provided information regarding effects of various diets (such as animal-based diet, plant-based diet, Mediterranean diet) and dietary components (such as fiber, resistant starch, polyphenols) in modifying gut microbiota composition. Reports regarding diet quality and its association with gut microbiota composition and metabolic functions are few (11–13). The evidence thus far indicates that a higher dietary quality associates with beneficial bacteria and its increased diversity (11, 12). Some indication also exists for the influence of diet with gut microbial functional benefit (13). This small (*n* =144) longitudinal study reported that, metabolic pathways related to an increased alpha-diversity were associated with habitual diet. However, the association between the host’s nutritional status, diet and gut microbiota remains to be elucidated further.

There are a myriad of mechanisms through which the gut microbiota may mediate human health effects. One of the key routes is considered to be low-grade inflammation, which is also observed in obesity (14, 15). A low bacterial gene richness has been associated with a more pronounced inflammatory phenotype (4). In our previous study with overweight and obese pregnant women, a higher gut microbiota richness defined by 16S rRNA gene sequencing was related to lower levels of GlycA, a novel marker of low-grade inflammation (16). As compared to 16S rRNA sequencing, the metagenomics approach provides a higher level of resolution at the strain- and species-level of microbiota as well as functional potential of microbiota (17), which yields further benefit in evaluation of the diet-microbiota-metabolism associations.

Here, our first aim was to investigate the association between dietary quality (18) and gut microbiota composition, analyzed by metagenomics. Our second aim was to examine the relationship between the composition of the gut microbiota and the metabolic and inflammatory status of the individual. We investigated whether the overweight and obese individuals would reveal distinct associations between dietary quality, gut microbiota composition and metabolic and inflammatory status. Finally, we also investigated the association between microbial functional pathways with dietary quality and body mass index (BMI) groups (overweight and obese).

## RESULTS

### Characteristics.

Baseline characteristics of the women and the values for dietary quality, gut microbiota diversity indices, and serum metabolic markers are presented in Table 1. The majority of the women (61%, 234 out of 386) were overweight, 39% were obese (152 out of 386), and 47% had a good dietary quality (IDQ score above 10.0). No difference in the IDQ score was seen between overweight (median [interquartile range; IQR] of 9.7 [8.0–11.0]) and obese women (median [IQR] of 9.7 [7.7–11.0]). Overall, the prepregnancy BMI was related to gut microbiota species diversity (i.e., the higher the BMI, the lower the species diversity; *r* = -0.147, *P *= 0.004). Top taxa observed included bacterial species *Clostridiales* sp., Bacteroides vulgatus, Bacteroides uniformis, *Clostridia* sp. and *Lachnospiraceae* sp. No significant differences in the relative abundances of bacterial taxa were observed between overweight and obese women.

**TABLE 1:** Baseline characteristics and concentrations of serum markers of glucose metabolism and low-grade inflammation, gut microbiota diversity and dietary index scores of the women^*a*^

Variables	All Women (*n* = 386)	Overweight (*n* = 234)	Obese (*n* = 152)	*P* ^*b*^
Age (yr)	30.5 (27.6–34.0)	30.3 (27.6–33.7)	30.8 (27.3–34.4)	0.40
University or college degree	56.5 (218/349)	56 (130/213)	42 (88/138)	0.58
Smoked before pregnancy	19.4 (75/351)	22 (51/213)	16 (24/138)	0.14
Dietary quality				
IDQ score	9.7 (8.0–11.0)	9.7 (8.0–11.0)	9.6 (7.7–11.0)	0.57
Good dietary quality	47 (182/386)	46 (108/234)	49 (74/152)	
Gut microbiota diversity^*c*^				
Species diversity	2.7 (2.5–2.9)	2.7 (2.5–2.9)	2.6 (2.4–2.8)	0.01
Species richness	237.0 (195.8–279.5)	241.5 (199.0–289.0)	231.0 (192.75–266.75)	0.02
Glucose metabolism				
Insulin (mU/l)	10.0 (7.0–13.0)	8.0 (7.0–10.75)	13.0 (9.0–17.0)	<0.001
Glucose (mmol/l)	4.7 (4.5–5.0)	4.7 (4.5–4.9)	4.8 (4.6–5.1)	0.002
QUICKI	0.3 (0.3–0.4)	0.35 (0.3–0.4)	0.33 (0.31–0.35)	<0.001
HOMA2-IR	1.3 (0.9–1.7)	1.0 (0.9–1.4)	1.6 (1.2–2.2)	<0.001
Low-grade inflammation				
GlycA (mmol/l)	1.2 (1.1–1.3)	1.2 (1.1–1.3)	1.2 (1.1–1.3)	<0.001
hsCRP (mg/l)	5.5 (3.2–8.9)	4.5 (2.6–7.2)	6.7 (4.0 – 10.7)	<0.001

a The values represent median (IQR) and percentages (Number of all or overweight or obese women).

b Group differences between overweight and obese women tested by Mann-Whitney U-test.

c Species diversity (Shannon index) and species richness were calculated with *vegan* package in R.

### Dietary quality in relation to gut microbiota diversity and serum markers.

Positive correlations with IDQ scores and species diversity (*r* = 0.273, *P *= 0.007) were detected in obese women, but not in their overweight counterparts (*r* = -0.039, *P *= 1).

Furthermore, when the dichotomized IDQ was evaluated (The quality of the diet was defined as poor when the score was less than 10 out of the maximum 15 points and good when points were 10 or more out of 15) (18), obese women with poor diet quality manifested a lower species diversity (median [IQR] species diversity = 2.60 [2.40–2.77]) compared to obese women with good dietary quality (median (IQR) species diversity = 2.68 [2.52–2.94], *P *= 0.03). No such similar association was detected in overweight women (poor dietary quality: median [IQR] species diversity = 2.74 (2.54–2.91); good dietary quality: median [IQR] species diversity = 2.69 [2.51–2.94], *P *= 0.51). No relationship between IDQ scores and beta diversity (permutational multivariate analysis of variance (PERMANOVA) analysis with Aitchison distance) was observed when all women were examined as a single group or when overweight and obese women were evaluated separately (Table 2).

**TABLE 2:** Associations between beta diversity (PERMANOVA) and diet quality (IDQ) and serum variables

Variables	All women			Overweight			Obese		
	Adj. *P*^*a*^	R2^*b*^	F^*c*^	Adj. *P*	R2	F	Adj. *P*	R2	F
IDQ	0.20	0.0039	1.504	1.00	0.0046	1.063	0.32	0.0094	1.436
Insulin	**0.04**	0.0057	2.225	0.24	0.0059	1.381	0.16	0.0101	1.537
HOMA	**0.04**	0.0059	2.309	0.36	0.0058	1.359	0.12	0.0105	1.606
GlycA	**0.04**	0.0066	2.562	**0.04**	0.0089	2.094	1.00	0.0069	1.054

a Adj.*P*: PERMANOVA *P*-value corrected for multiple testing. Adjusted *p*-value < 0.05 was considered significant (marked as bold).

b R2-value for PERMANOVA.

c F Value for PERMANOVA.

The association between dichotomized IDQ and beta diversity was also evaluated but no significant associations were observed for all (*P *= 0.10) or obese women (*P *= 0.38), although borderline significance was observed for overweight women (*P *= 0.09).

An inverse correlation was observed between dietary IDQ scores and GlycA in all women (*r* = -0.162, *P *= 0.007) and in obese women (*r* = -0.222, *P *= 0.04), but not in overweight women (*r* = -0.125, *P *= 0.39). No such associations were observed with hsCRP or in the other examined metabolic markers either in all pregnant women or in the overweight and obese subgroups (data not shown).

### Gut microbiota in relation to metabolic and inflammatory markers.

First, when all women were analyzed together, a borderline significant finding was detected for the relation between gut microbiota species diversity and markers of glucose metabolism and low-grade inflammation. Namely, the lowest quartile of species diversity was related to higher concentrations of insulin (*P *= 0.07) and HOMA2-IR (homeostasis model assessment 2- insulin resistance) (*P *= 0.08) and lower QUICKI (quantitative insulin sensitivity check index) (*P *= 0.11) compared to the highest quartile, but no relationship was evident with glucose levels (*P *= 1.0) (Table 3). In addition, in the lowest quartile of species diversity, the level of the novel inflammatory marker, GlycA, was higher compared to the highest quartile (*P *= 0.07), but the more traditional marker of low-grade inflammation, hsCRP was not related with the species diversity index (*P *= 1.0) (Table 3). With higher insulin (*P *= 0.01) and GlycA levels (*P *< 0.001), and HOMA2-IR (*P *= 0.006), but lower QUICKI (*P *= 0.02), compared to the highest quartile (Table 3).

**TABLE 3:** Differences in markers of glucose metabolism and low-grade inflammation between the highest (Q4) and the lowest (Q1) gut microbiota diversity and richness indices^*a*^

	All Women			Overweight			Obese			
			Q1 vs Q4			Q1 vs Q4				Q1 vs Q4
Variables	Q1	Q4	Adj. *P*^*b*^	Q1	Q4	Adj. *P*		Q1	Q4	Adj. *P*
Species diversity^*b*^	*n* = 94; 2.32 (2.19–2.40)	*n* = 93; 3.01 (2.96–3.07)		*n* = 52; 2.33 (2.23–2.42)	*n* = 65; 3.01 (2.96–3.06)			*n* = 42; 2.28 (2.14–2.40)	*n* = 28; 3.0 (2.96–3.11)	
Insulin mU/l	10.0 (7.0–15.0)	8.0 (7.0–11.5)	**0.07**	9.0 (7.0–11.75)	8.0 (6.0–9.5)	0.29		13.5 (8.75–19.0)	12.0 (8.0–16.75)	1.0
Glucose mmol/l	4.7 (4.6–5.0)	4.7 (4.5–5.0)	1.0	4.6 (4.5–4.9)	4.7 (4.5–4.9)	1.0		4.9 (4.6–5.2)	4.8 (4.5–5.08)	1.0
HOMA2 IR	1.3 (0.9–1.9)	1.0 (0.9–1.5)	**0.08**	1.1 (0.9–1.47)	1.0 (0.80–1.25)	0.39		1.7 (1.08–2.43)	1.5 (1.03–2.1)	1.0
QUICKI	0.34 (0.32–0.36)	0.35 (0.33–0.36)	0.11	0.35 (0.33–0.36)	0.35 (0.34–0.37)	0.47		0.33 (0.31–0.35)	0.33 (0.31–0.35)	1.0
hsCRP mg/l	5.1 (3.1–8.8)	4.6 (3.0–7.7)	1.0	4.35 (2.65–6.48)	3.9 (2.4–6.5)	1.0		6.95 (4.0–11.03)	6.45 (4.30–8.93)	1.0
GlycA mmol/l	1.22 (1.15–1.29)	1.17 (1.11–1.27)	**0.07**	1.21 (1.13–1.27)	1.15 (1.09–1.25)	0.28		1.25 (1.18–1.34)	1.24 (1.16–1.30)	1.0
									
Species richness^*c*^	*n* = 96; 174 (157–186)	*n* = 96; 307 (294–328)		*n* = 55; 177 (162–186)	*n* = 70; 308.5 (294–328.3)			*n* = 41; 169 (149–184.5)	*n* = 26; 304 (294.8– 327)	
Insulin mU/l	10.0 (8.0–15.0)	8.0 (7.0–11.0)	**0.01**	10 (8.0–11.0)	8.0 (6.75–9.0)	**<0.001**		13.0 (8.0–18.0)	14.0 (10.0–16.0)	1.0
Glucose mmol/l	4.8 (4.6–5.1)	4.7 (4.4–4.9)	0.38	4.6 (4.5–4.9)	4.6 (4.4–4.9)	1.0		4.9 (4.65–5.2)	4.8 (4.48–5.0)	1.0
HOMA2 IR	1.3 (1.0–2.0)	1.0 (0.9–1.4)	**0.006**	1.3 (1.0–1.5)	1.0 (0.88–1.13)	**<0.001**		1.6 (1.0–2.3)	1.8 (1.2–2.03)	1.0
QUICKI	0.34 (0.32–0.35)	0.35 (0.33–0.36)	**0.02**	0.34 (0.33–0.36)	0.35 (0.35–0.36)	**<0.001**		0.33 (0.31–0.35)	0.32 (0.32–0.35)	1.0
hsCRP mg/l	5.9 (3.3–9.7)	4.6 (2.6–7.0)	**0.09**	5.5 (3.0–9.2)	4.25 (2.15–6.13)	0.11		6.3 (3.8–9.95)	5.75 (1.16–1.29)	1.0
GlycA mmol/l	1.23 (1.2–1.31)	1.16 (1.10–1.26)	**<0.001**	1.22 (1.16–1.28)	1.15 (1.09–1.24)	**<0.001**		1.24 (1.17–1.36)	1.22 (1.16–1.29)	1.0

a The values represent median (IQR). Mann-Whitney U test for differences between the highest and the lowest gut microbiota diversity quartiles.

b Multiple testing corrected *P*-value. Adjusted *P*-value < 0.05 was considered significant; adjusted *P*-value < 0.1 was considered borderline significant (both marked as bold).

c Species diversity (Shannon index) and species richness were calculated with *vegan* package in R.

When the women were subdivided into either overweight or obese, no relation was found between quartiles of species diversity and any of the serum metabolic and inflammatory markers (Table 3). The same was found when the correlation between gut microbiota species diversity and the markers mentioned above were inspected (Table 4). However, the lowest quartile of species richness was associated with higher insulin, HOMA2-IR, and GlycA and lower QUICKI (all, *P* < 0.001) only in overweight women. Similar results were observed with the correlation between gut microbiota species richness and insulin (*P *= 0.006), HOMA2-IR (*P *= 0.01), QUICKI (*P *= 0.02), and GlycA (*P *< 0.001) (Tables 3 and 4).

**TABLE 4:** Correlations between gut microbiota diversity and richness indices and glucose metabolism and low-grade inflammation in all women as well as in overweight and obese women

Variables	Insulin		Glucose		HOMA2-IR		QUICKI		hsCRP		GlycA	
All women	r^*a*^	Adj. *P*^*b*^	r	Adj. *P*	r	Adj. *P*	r	Adj. *P*	r	Adj. *P*	r	Adj. *P*
Species diversity^*c*^	-0.145	**0.02**	-0.75	0.75	-0.144	**0.03**	0.141	**0.03**	-0.086	0.55	-0.123	**0.09**
Species richness^*c*^	-0.166	**0.006**	-0.105	0.23	-0.168	**<0.001**	0.161	**0.01**	-0.112	0.16	-0.235	**<0.001**
Overweight	r	Adj. *P*	r	Adj. *P*	r	Adj. *P*	r	Adj. *P*	r	Adj. *P*	r	Adj. *P*
Species diversity	-0.151	0.12	-0.013	1.0	-0.145	0.16	0.139	0.198	-0.061	1.0	-0.131	0.27
Species richness	-0.0210	**0.006**	-0.055	1.0	-0.205	**0.01**	0.192	**0.02**	-0.141	0.13	-0.273	**<0.001**
Obese	r	Adj. *P*	r	Adj. *P*	r	Adj. *P*	r	Adj. *P*	r	Adj. *P*	r	Adj. *P*
Species diversity	-0.048	1.0	-0.128	0.70	-0.055	1.0	0.049	1.0	-0.065	1.0	-0.027	1.0
Species richness	-0.030	1.0	-0.140	0.51	-0.041	1.0	0.041	1.0	-0.001	1.0	-0.111	1.0

a *r* = Spearman’s correlation coefficient.

b Adj. *P *: multiple testing corrected *P*-value from Spearman’s correlation analysis. Adjusted *P*-value < 0.05 was considered significant; Adjusted *P*-value < 0.1 was considered borderline significant (both marked as bold).

c Species diversity (Shannon index) and species richness were calculated with *vegan* package in *R*.

In addition, we examined the association between the community composition (beta diversity) and pre-selected serum metabolic and inflammatory markers. Beta diversity was associated with serum metabolism markers i.e., insulin, HOMA2-IR and GlycA (PERMANOVA analysis with Aitchison distance, *P* = 0.04, in all cases) (Table 2). When overweight and obese women were analyzed separately, the GlycA level was found to be associated with beta diversity in overweight (*P *= 0.04), but not in obese women. No such associations were observed between beta diversity and other serum markers (i.e., insulin and HOMA2-IR). Due to the association between beta diversity and GlycA, we further investigated whether any specific bacterial species would be related to GlycA. We found a positive correlation between Alistipes finegoldii abundance and GlycA levels in overweight (*P *= 0.03), but not in obese women (*P *= 0.99).

### Functional analysis.

When associations between IDQ and 195 KEGG pathways were investigated, we observed one pathway, Iron complex transport system (M00240) that showed borderline negative correlation (Spearman rho = −0.18, *P *= 0.09) with IDQ score. No other statistically significant associations were observed (data not shown). Also, no statistically significant associations were observed between overweight or obese BMI groups and KEGG pathways (data not shown).

## DISCUSSION

**FIG 1:**
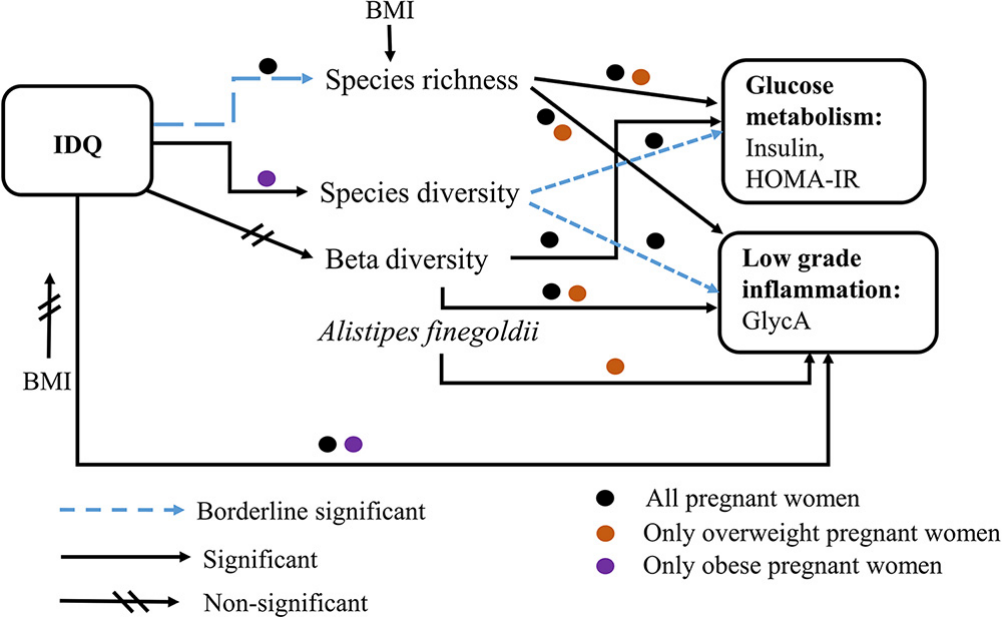
Summary of the findings related to associations between diet quality, alpha and beta diversity, gut microbiota, and serum markers in the overweight and obese women. In overweight women, a higher gut microbiota species richness correlates with glucose metabolism and low-grade inflammatory markers, whereas dietary quality does not associate with either species richness or species diversity (Shannon index), the indices for alpha diversity. Further, there are correlations between inflammation marker and beta diversity as well as with one bacterium species Alistipes finegoldii in only overweight women. Although the species diversity of obese women seems to be linked with higher dietary quality, this is not reflected in maternal metabolism and inflammatory status. Instead, in obese women, dietary quality correlated with maternal inflammatory marker. When all women were studied together, beta diversity associated with glucose metabolism markers. In addition, borderline significant associations were observed between species diversity and markers of glucose metabolism and inflammation. Another index, species richness was significantly associated with these markers. BMI: body mass index; IDQ: index of diet quality. Black dots indicate all women, orange dots represent only the overweight pregnant women and purple dots refer to only obese pregnant women. Associations are indicated as- solid black arrow: significant (*P *< 0.05), solid black arrow with two crossed lines: non-significant (*P *≥ 0.1) and dashed blue arrow: borderline significant (*P *< 0.1) (*P*: multiple testing corrected *P*-values).

We demonstrated here that there are partially distinct associations between microbiota, diet quality, and metabolic and inflammatory markers in overweight and obese pregnant women (please see a summary in Fig. 1). Our findings suggest that the metabolic and inflammatory profiles of overweight women benefit from gut microbiota species richness and overall bacterial composition (beta diversity), but in obese women, the quality of diet may influence species diversity and modify the inflammatory profile, particularly the GlycA level.

Our findings show that the diversity of gut microbiota species reflects dietary quality in obese women in early pregnancy. As far as we are aware, this is the first study to have applied a metagenomics approach to explore the relationship between dietary quality and gut microbiota diversity, richness and function in pregnancy. The metagenomics approach provides a higher level of resolution at the strain- and species-level of microbiota compared to previous studies using 16S rRNA gene sequencing. Metagenomics allows us to explore more specific taxonomic and functional classifications for the microbial community (17). In previous studies, two dietary measures, described as the healthy eating index (compliance with the U.S. Dietary Guideline for Americans) and the Mediterranean diet score (adherence to a Mediterranean diet) correlated with gut microbiota diversity indices in non-pregnant subjects (19). In another study in women with gestational diabetes, no differences were observed in gut microbiota diversity between the women who adhered to the dietary recommendations and those not eating according to these guidelines (20). In our smaller scale study of 84 women, the dietary quality correlated positively with gut microbiota diversity (11). Unlike the previous studies, here we differentiated the participants according to their overweight and obesity status. Indeed, if the women were classified as either overweight or obese, this altered the correlation between dietary quality and gut microbiota species diversity (i.e., the relationship was only observed in obese women). The reason for this observation remains unclear. The dietary quality *per se* did not differ between overweight and obese women, but the result suggests that the gut microbiota of obese individuals is more adaptable to dietary modifications.

Interestingly, species richness and community composition were related to both the metabolic and inflammatory profiles, but only in the overweight women. An inverse correlation was observed between the gut microbiota species richness and markers of glucose metabolism and inflammation, here assessed via the GlycA level. Further, the community composition was associated with the GlycA level. In the taxonomic analysis, only one association (i.e., a positive association was found between GlycA and *A. finegoldii* and only in overweight women). *A. finegoldii* is a bile resistant bacterium, and lower counts have been found in patients with nonalcoholic steatohepatitis (NASH) when compared to healthy adults (21), and a higher abundance has been associated with elevated blood pressure (22). GlycA is a novel marker for low grade inflammation which has been associated with metabolic complications including incident type 2 diabetes and cardiovascular diseases (23, 24). The traditional marker, hsCRP, was related to neither of the richness indices. In a previous study with a subset of the same study population, where 16S rRNA gene sequencing was utilized to explore gut microbiota alpha diversity, a negative correlation was found with GlycA in a combined group of overweight and obese women (16). Another study in normal weight, overweight, or obese pregnant women detected a negative correlation between the species diversity index and the level of hsCRP in the third trimester (7), but unlike in our current study, the overweight and obesity status was not considered. Nonetheless, as the metabolic burden is increased in association with maternal BMI (25), and in obese women compared to overweight women (26), we propose that a higher gut microbiota species richness may benefit the overweight women by alleviating disturbances in glucose metabolism and reducing the extent of low-grade inflammation. On the contrary, this was not observed in obese women; in these individuals, a higher IDQ was related to higher species diversity, indicating that the obese women with a more disturbed metabolic phenotype may benefit from dietary modification. This is evidenced by the finding of our study; the dietary quality in obese women was reflected in the maternal inflammatory status, and this relationship was independent of gut microbiota taxonomic diversity, but rather it seemed to originate from the direct effects of the diet on the mother’s health.

We also explored associations of diet quality and the BMI groups (overweight and obese) with gut microbiota functional potential. Although such associations might exist (11–13), we did not find any statistically significant associations. We observed one pathway, iron complex transport system that showed borderline negative correlation with diet quality, but its exact role is not yet understood. Reports investigating the relationships between diet quality and gut microbial function are all in all few (11–13).

Our study has several strengths. Firstly, we applied high resolution (i.e., at species-level) metagenomics analytics to assay the gut microbiota’s richness and diversity as well as performed functional analyses. Secondly, we used a validated method (18), for analyzing the dietary quality. Furthermore, due to the well-known effects of adiposity on dietary intake, gut microbiota and circulating metabolic profile, in this comprehensive approach, we took into account the possible influence of the prepregnancy BMI value. Another strength was that our study population was homogeneous with a similar age range and gender. The robustness of statistical analyses was ensured by applying multiple corrections. There are also limitations in our study. Even though the physiological alterations are mild in early pregnancy, it is not clear whether the study results are generalizable to non-pregnant conditions. It also needs to be clarified whether women in late pregnancy or normal weight pregnant subjects behave similarly. Although no statistically significant associations with species diversity and serum markers were observed, the complementary analyses using species richness indicate that such associations might well exist. These indices are complementary indicators and focus on the different aspects of the microbial community. These associations need to be confirmed in larger studies to determine the clear effects. In our study the group of obese women was smaller than that of overweight women which might limit observing statistically significant differences.

### Conclusions.

Our results demonstrate that overweight and obese women may display partially distinct metabolic and inflammatory responses to the diet and that they exhibit differences in their gut microbiota species richness and diversity, and community composition. A higher dietary quality was reflected in a richer gut microbiota species diversity, but only in obese women. In overweight women, a higher gut microbiota species richness was related to lower levels of markers of glucose metabolism and both species richness and community composition and one species (i.e., A. finegoldii) associated with low grade inflammation. The different responses to diet and gut microbiota species richness and diversity observed in overweight and obese women may originate from the differences in the metabolic and inflammatory burden related to the excess adiposity. The fact that we observed no relationship between metabolic and inflammatory status with gut microbiota species diversity in obese women suggests that other factors, such as diet as observed in our study, could have regulatory effects of maternal metabolism and inflammation in obese women, although the findings will need to be verified in larger studies. We also propose that the dietary quality index may be used as a tool for controlling the dietary intake in gut microbiota studies, but at the same time, one needs to consider the adiposity of the participants.

## MATERIALS AND METHODS

This was a cross-sectional study investigating overweight and obese pregnant women participating in a mother-infant dietary single-center intervention trial (ClinicalTrials.gov, NCT01922791) being conducted in Southwest Finland. The recruitment took place between 10/2013 and 7/2017. The inclusion criteria for the study were overweight (self-reported prepregnancy BMI ≥ 25 kg/m^2^) and early pregnancy (<18 weeks of gestation). The exclusion criteria were gestational diabetes diagnosed during the current pregnancy, multifetal pregnancy, and the presence of metabolic or inflammatory diseases, including type 1 and type 2 diabetes, celiac disease, and inflammatory bowel disease. The presence of allergy was allowed. Here we describe the interaction between dietary quality, fecal gut microbiota alpha diversity (species richness and Shannon diversity index), community composition i.e., beta diversity and species abundance, and serum markers of glucose metabolism and low-grade inflammation at baseline prior to the onset of the intervention. Women who had consumed antibiotics within 8 weeks before the study visit, and those who did not provide fecal or serum samples and did not fill in the dietary questionnaire were excluded, resulting in 386 out of 439 women being included in the analyses.

Prepregnancy BMI (kg/m^2^) was calculated by dividing self-reported weight in kilograms, obtained from welfare women clinic records, by height measured in the study visit with a wall stadiometer to the nearest 0.1 cm. Other characteristics of the women (Table 1), including age, education and smoking were collected by interviews and questionnaires.

This study was conducted according to the guidelines laid down in the Declaration of Helsinki as revised in 2013, and all procedures that involved human subjects were approved by the Ethics Committee of the Hospital District of Southwest Finland (permission number 115/180/2012). All participants provided written informed consent. The metadata are not publicly available due to their containing information that could compromise the privacy of research participants.

### Quality of the diet.

The overall dietary quality with reference to that recommended was measured with the validated index of diet quality (IDQ) questionnaire (18), which contains 18 questions regarding the frequency and amount of consumption of foods during the week preceding the study visit. The criteria for a health-promoting diet were derived from dietary recommendations and consisted of consumption of whole grain, vegetables, fruits and berries, dairy and choice of foods that will yield a good quality of dietary fat intake. The quality of the diet was defined as poor when index points were less than 10 out of the maximum 15 points and good when points were 10 or more out of 15 (18). Here, the IDQ score was used both as a continuous variable and as dichotomized in accordance with the previous article describing score (18).

### DNA extraction.

Fecal samples were collected in sterile plastic pots on the morning of the study visit or the previous evening, delivered to the study unit and kept at −20°C until DNA extraction. DNA was extracted from 50 mg of homogenized feces using GTX stool extraction kit and fully automated GenoXTract machine (Hain Lifescience, Nehren, Germany) as previously described (27). Prior to extraction, mechanical lysis was performed by bead-beating the samples in ceramic bead tubes with MOBIO PowerLyzerTM 24 Bench Top Bead-Based Homogenizer (MO BIO Laboratories, Inc., USA). The DNA concentrations were measured with Qubit 2.0 dsDNA HS assay kit (Life Technologies), after which the DNAs were stored at −80°C until sequencing.

### Metagenomic sequencing.

The genomic DNA was randomly sheared into fragments of approximately 350 bp. The fragmented DNA was used for library construction using NEBNext Ultra II Library Prep Kit for Illumina (New England Biolabs). The prepared DNA libraries were evaluated using Qubit 2.0 fluorometer quantitation and Agilent 2100 Bioanalyzer for the fragment size distribution. Quantitative real-time PCR (qPCR) was used to determine the concentration of the final library before sequencing. The library was sequenced using 2 × 150 bp paired-end sequencing on an Illumina HiSeq platform.

### Bioinformatics processing.

**(i) Data preprocessing.** The raw FASTQ files were quality controlled using KneadData (v.0.6.1) to remove low-quality bases and reads derived from the host genome as follows: Using Trimmomatic (v.0.36), the reads were quality trimmed by removing Nextera adapters, leading or trailing bases with a Phred score below 20, and trailing bases in which the Phred score over a window of size 4 drops below 20. Trimmed reads shorter than 100 bases were discarded. Reads that mapped to the human reference genome GRCh38 (with Bowtie2 v.0.2.3.2 using default settings) were also discarded. Read pairs in which both reads passed filtering were retained; these were classified as high-quality non-host (HQNH) reads.

**(ii) Mapping reads to gene catalog.** HQNH reads were mapped to the integrated gene catalog (IGC) (28), using BWA mem (v.0.7.16a) with options to increase accuracy (-r 1 -D 0.3). PCR/optical duplicates were removed using samtools (v.1.6). A read pair where both reads had a mapping quality (MAPQ) ≥ 20 and an alignment of at least 100 bp and with ≥ 95% identity to a single IGC gene was considered mapped. However, the mapping was rejected if > 10 bases at either end of the read failed to align to an existing gene sequence (i.e., alignment beyond the IGC gene sequence was accepted). The read counts were used to estimate the abundance of the Clinical Microbiomics proprietary set of IGC metagenomic species (MGS) (29) derived from abundance profiles across 3200 reference samples. For each MGS, the “core” genes have been defined as the 100 genes with the highest correlation of abundance across the reference samples. A table of MGS counts was created based on the total gene counts for the 100 core genes of each MGS. However, an MGS was considered detected only if the read pairs were mapped to at least three of the 100 core genes; MGSs that did not satisfy this criterion were set as zero counts. The relative abundance estimate of each MGS was made by normalizing the counts for gene lengths. Rarefied (downsampled) MGS abundance profiles were calculated by performing the above procedure on a rarefied gene counts table (generated by random sampling, without replacement, of HQNH read pairs).

The species diversity (Shannon index) and richness were calculated with *vegan R* package (30), from the number of metagenomics species (MGSs) that were detected and their relative abundances in the rarified (7,281,907 read pairs) data. Richness describes the number of species that is detected in a sample, whereas the Shannon diversity index also takes the relative abundance of the species into account. Shannon diversity is less sensitive to sampling errors than species richness, as it gives a higher weight to the more abundant species than to their rarer counterparts.

**(iii) Functional annotation of gene catalog.** Emapper software (v. 1.0.3, HMM mode) was used to compare each gene in the gene catalog to the EggNOG (v. 4.5) orthologous groups database (http://eggnogdb.embl.de/). These genes were then mapped from EggNOG to Kyoto Encyclopedia of Genes and Genomes (KEGG) orthologies (KO) and modules (http://www.genome.jp/kegg/kegg1.html) using MOCAT2 lookup tables (http://mocat.embl.de/). KEGG pathways that had > 40% prevalence (i.e., pathways which were observed in more than 40% of study population) were selected. Altogether, 195 KEGG pathways were selected for microbial functional analyses out of a total of 530 pathways present in the data.

### Sequencing statistics.

On average about 21% reads were discarded being low quality, 0.5% for mapped to the host genome, and 10% for not mapping to the gene catalog, leaving about 69% mapped reads (Table S1 in the supplemental material). Information about read counts (%) per sample is provided as a figure (Fig. S1).

### Taxonomy reads annotation.

Reads with an unknown taxonomy were reported as unmapped (if reads not mapped to the gene catalog) and orphan genes (if mapped to the gene catalog but not attributed to an MGS). Mean relative abundances (%) were calculated for bacterial taxa for overweight and obese (Table S2). The differences in the relative abundances of bacterial taxa between overweight and obese were analyzed with Mann-Whitney test and were corrected with Benjamini-Hochberg method. Adjusted *P*-value < 0.05 was considered as significant.

### Taxonomic and functional analyses.

Only those serum variables, which had significant associations with species richness i.e., insulin, HOMA2-IR and GlycA were selected for beta diversity (community composition) analysis. Associations between beta diversity and these continuous variables as well as association between beta diversity and dichotomized IDQ were quantified with PERMANOVA using the *adonis* function from the *vegan* R package. For beta diversity analysis reads were not rarefied. PERMANOVA was used to test the significance of association based on the Aitchison distance (centered log ratio (CLR) transformation of abundance with Euclidean distance); the *P*-values were adjusted with the Bonferroni method. We quantified the association between the GlycA level and individual taxonomic groups (bacterial species) with DESeq2 (31), separately for overweight and obese women. For DESeq2 analysis, original count data was used. The multiple hypothesis correction was done with Bonferroni method. Adjusted *P*-value < 0.05 was considered as significant, whereas cases with adjusted *P*-value < 0.1 were considered to be borderline significant. Associations of KEGG pathways with diet quality index (IDQ) and both BMI groups (overweight and obese) were quantified with Spearman correlation and Wilcoxon test, respectively. The multiple hypothesis correction was done with Benjamini-Hochberg method. The taxonomic and functional analyses were performed in R (v.3.6.3).

### Markers of glucose metabolism and low-grade inflammation.

Fasting (10 h’ minimum) blood samples were drawn from the antecubital vein, and the serum was separated and analyzed for insulin, glucose, and hsCRP, and the rest of the samples were frozen in aliquots at −80°C until being analyzed for serum metabolomics. A high-throughput proton NMR metabolomics platform (Nightingale, Helsinki, Finland) was used to analyze (32), the level of serum glycoprotein acetylation (GlycA), a novel marker of low-grade inflammation. GlycA consists of a complex heterogeneous nuclear magnetic resonance signal originating from the N-acetyl sugar groups present on multiple acute phase glycoproteins in the circulation; α1-acid glycoprotein, haptoglobin, α1-antitrypsin, α1-antichymotrypsin and transferrin (33). The concentrations of glucose, insulin, and hsCRP were measured in an accredited Turku University Hospital Laboratory according to its quality control system. The glucose concentration was measured using an enzymatic method utilizing hexokinase (Cobas 8000 automatic c702-analyzer, Roche Diagnostics GmbH, Mannheim, Germany). Insulin concentrations were determined with an immunoelectrochemiluminometric assay (a modular E170 automatic analyzer, Roche Diagnostics GMbH, Mannheim, Germany). Insulin resistance, HOMA2-IR, was calculated from fasting plasma glucose and fasting insulin using HOMA calculator (http://www.dtu.ox.ac.uk/). Insulin sensitivity, QUICKI, was calculated as = 1/[log(FastingInsulin) + log(FastingGlucose)] (34). HsCRP levels were determined using an automated colorimetric immunoassay on the Dade Behring Dimension RXL autoanalyzer (Siemens Healthcare, Camberly, Surrey, UK). The lower limit of detection was 0.1 mg/L.

### Statistics.

As not all variables were normally distributed (inspected using histograms and Kolmogorov-Smirnov test), non-parametric tests were used for all statistical tests. The relationship of IDQ and prepregnancy BMI with gut microbiota species richness and diversity with markers of glucose metabolism and low-grade inflammation was analyzed by Spearman’s correlation. The correlations of species richness and diversity with IDQ and serum variables were corrected for multiple testing. The differences in gut microbiota species richness and diversity between the highest and the lowest quartiles of IDQ, between the poor (IDQ < 10) and the good (IDQ ≥ 10) dietary quality and between overweight (prepregnancy BMI < 30) and obese status (prepregnancy BMI ≥ 30) and group difference between overweight and obese for baseline characteristics, were investigated using Mann-Whitney U test. The differences in markers of glucose metabolism and low-grade inflammation between the highest and the lowest species richness and diversity quartile were analyzed by Mann-Whitney U test. The *P*-values obtained were corrected for multiple testing. Statistical analyses were performed with SPSS for Windows, version 24 (IBM Corp., Armonk, NY) with adjusted *P* < 0.05 considered statistically significant, whereas cases with adjusted *P*-value < 0.1 were considered to be borderline significant.

### Data availability.

The data sets are not available due to their containing information that could compromise participant privacy and consent.

## References

[1] Mokkala K, Houttu N, Cansev T, Laitinen K. 2020. Interactions of dietary fat with the gut microbiota: Evaluation of mechanisms and metabolic consequences. Clin Nutr 39:994–1018. doi:10.1016/j.clnu.2019.05.003. 31171289

[2] O’Keefe SJ, Li JV, Lahti LM, Ou J, Carbonero F, Mohammed K, Posma JM, Kinross J, Wahl E, Ruder E, Vipperla K, Naidoo V, Mtshali L, Tims S, Puylaert PGB, DeLany J, Krasinskas A, Benefiel AC, Kaseb HO, Newton K, Nicholson JK, de Vos WM, Gaskins HR, Zoetendal EG. 2015. Fat, fibre and cancer risk in African Americans and rural Africans. Nat Commun 6:6342. doi:10.1038/ncomms7342. 25919227 PMC4415091

[3] Rowland I, Gibson G, Heinken A, Scott K, Swann J, Thiele I, Tuohy K. 2018. Gut microbiota functions: metabolism of nutrients and other food components. Eur J Nutr 57:1–24. doi:10.1007/s00394-017-1445-8. PMC5847071 28393285

[4] Le Chatelier E, Nielsen T, Qin J, Prifti E, Hildebrand F, Falony G, Almeida M, Arumugam M, Batto JM, Kennedy S, Leonard P, Li J, Burgdorf K, Grarup N, Jørgensen T, Brandslund I, Nielsen HB, Juncker AS, Bertalan M, Levenez F, Pons N, Rasmussen S, Sunagawa S, Tap J, Tims S, Zoetendal EG, Brunak S, Clément K, Doré J, Kleerebezem M, Kristiansen K, Renault P, Sicheritz-Ponten T, De Vos WM, Zucker JD, Raes J, Hansen T, Bork P, Wang J, Ehrlich SD, Pedersen O, Guedon E, Delorme C, Layec S, Khaci G, Van De Guchte M, Vandemeulebrouck G, Jamet A, Dervyn R, Sanchez N, MetaHIT consortium, et al. 2013. Richness of human gut microbiome correlates with metabolic markers. Nature 500:541–546. doi:10.1038/nature12506. 23985870

[5] Schwingshackl L, Bogensberger B, Hoffmann G. 2018. Diet quality as assessed by the healthy eating index, alternate healthy eating index, dietary approaches to stop hypertension score, and health outcomes: an updated systematic review and meta-analysis of cohort studies. J Acad Nutr Diet 118:74–100.e11. doi:10.1016/j.jand.2017.08.024. 29111090

[6] Vinke PC, Navis G, Kromhout D, Corpeleijn E. 2021. Associations of diet quality and all-cause mortality across levels of cardiometabolic health and disease: a 7.6-year prospective analysis from the Dutch Lifelines Cohort. Dia Care 44:1228–1235. doi:10.2337/dc20-2709. 33963020

[7] Zacarías MF, Collado MC, Gómez-Gallego C, Flinck H, Aittoniemi J, Isolauri E, Salminen S. 2018. Pregestational overweight and obesity are associated with differences in gut microbiota composition and systemic inflammation in the third trimester. PLoS One 13:e0200305. doi:10.1371/journal.pone.0200305. 30005082 PMC6044541

[8] Aoun A, Darwish F, Hamod N. 2020. Faculty of Nursing and Health Sciences, Notre Dame University-Louaize, Zouk Mosbeh 72. Prev Nutr Food Sci 25:113–123. doi:10.3746/pnf.2020.25.2.113. 32676461 PMC7333005

[9] Paoli A, Mancin L, Bianco A, Thomas E, Mota JF, Piccini F. 2019. Ketogenic diet and microbiota: friends or enemies? Genes 10:534. doi:10.3390/genes10070534. 31311141 PMC6678592

[10] Beam A, Clinger E, Hao L. 2021. Effect of diet and dietary components on the composition of the gut microbiota. Nutrients 13:2795. doi:10.3390/nu13082795. 34444955 PMC8398149

[11] Laitinen K, Mokkala K. 2019. Overall dietary quality relates to gut microbiota diversity and abundance. Int J Mol Sci 20:1835. doi:10.3390/ijms20081835. 31013927 PMC6515207

[12] Liu Y, Ajami NJ, El-Serag HB, Hair C, Graham DY, White DL, Chen L, Wang Z, Plew S, Kramer J, Cole R, Hernaez R, Hou J, Husain N, Jarbrink-Sehgal ME, Kanwal F, Ketwaroo G, Natarajan Y, Shah R, Velez M, Mallepally N, Petrosino JF, Jiao L. 2019. Dietary quality and the colonic mucosa-associated gut microbiome in humans. Am J Clin Nutr 110:701–712. doi:10.1093/ajcn/nqz139. 31291462 PMC6736447

[13] Yu D, Yang Y, Long J, Xu W, Cai Q, Wu J, Cai H, Zheng W, Shu XO. 2021. Long-term diet quality and gut microbiome functionality: a prospective, shotgun metagenomic study among urban Chinese adults. Curr Dev Nutr 5:1–8. 33937616 10.1093/cdn/nzab026 PMC8068758

[14] van den Munckhof ICL, Kurilshikov A, ter Horst R, Riksen NP, Joosten LAB, Zhernakova A, Fu J, Keating ST, Netea MG, de Graaf J, Rutten JHW. 2018. Role of gut microbiota in chronic low-grade inflammation as potential driver for atherosclerotic cardiovascular disease: a systematic review of human studies. Obes Rev 19:1719–1734. doi:10.1111/obr.12750. 30144260

[15] Ferreira CM, Vieira AT, Vinolo MAR, Oliveira FA, Curi R, Martins F.dS. 2014. The central role of the gut microbiota in chronic inflammatory diseases. J Immunol Res 2014:689492. doi:10.1155/2014/689492. 25309932 PMC4189530

[16] Röytiö H, Mokkala K, Vahlberg T, Laitinen K. 2017. Dietary intake of fat and fibre according to reference values relates to higher gut microbiota richness in overweight pregnant women. Br J Nutr 118:343–352. doi:10.1017/S0007114517002100. 28901891

[17] Jovel J, Patterson J, Wang W, Hotte N, O’Keefe S, Mitchel T, Perry T, Kao D, Mason AL, Madsen KL, Wong GKS. 2016. Characterization of the gut microbiome using 16S or shotgun metagenomics. Front Microbiol 7:1–17. 27148170 10.3389/fmicb.2016.00459 PMC4837688

[18] Leppälä J, Lagström H, Kaljonen A, Laitinen K. 2010. Construction and evaluation of a self-contained index for assessment of diet quality. Scand J Public Health 38:794–802. doi:10.1177/1403494810382476. 20846997

[19] Bowyer RCE, Jackson MA, Pallister T, Skinner J, Spector TD, Welch AA, Steves CJ. 2018. Use of dietary indices to control for diet in human gut microbiota studies. Microbiome Springer Science and Business Media LLC 6 doi:10.1186/s40168-018-0455-y. PMC5918560 29695307

[20] Ferrocino I, Ponzo V, Gambino R, Zarovska A, Leone F, Monzeglio C, Goitre I, Rosato R, Romano A, Grassi G, Broglio F, Cassader M, Cocolin L, Bo S. 2018. Changes in the gut microbiota composition during pregnancy in patients with gestational diabetes mellitus (GDM). Sci Rep 8:12216–12216. doi:10.1038/s41598-018-30735-9. 30111822 PMC6093919

[21] Rau M, Rehman A, Dittrich M, Groen AK, Hermanns HM, Seyfried F, Beyersdorf N, Dandekar T, Rosenstiel P, Geier A. 2018. Fecal SCFAs and SCFA-producing bacteria in gut microbiome of human NAFLD as a putative link to systemic T-cell activation and advanced disease. United European Gastroenterol J 6:1496–1507. doi:10.1177/2050640618804444. PMC6297934 30574320

[22] Kim S, Goel R, Kumar A, Qi Y, Lobaton G, Hosaka K, Mohammed M, Handberg EM, Richards EM, Pepine CJ, Raizada MK. 2018. Imbalance of gut microbiome and intestinal epithelial barrier dysfunction in patients with high blood pressure. Clin Sci 132:701–718. doi:10.1042/CS20180087. PMC5955695 29507058

[23] Connelly MA, Gruppen EG, Wolak-Dinsmore J, Matyus SP, Riphagen IJ, Shalaurova I, Bakker SJL, Otvos JD, Dullaart RPF. 2016. GlycA, a marker of acute phase glycoproteins, and the risk of incident type 2 diabetes mellitus: PREVEND study. Clin Chim Acta 452:10–17. doi:10.1016/j.cca.2015.11.001. 26549655

[24] Connelly MA, Otvos JD, Shalaurova I, Playford MP, Mehta NN. 2017. GlycA, a novel biomarker of systemic inflammation and cardiovascular disease risk. J Transl Med 15:219. doi:10.1186/s12967-017-1321-6. 29078787 PMC5658936

[25] Sandler V, Reisetter AC, Bain JR, Muehlbauer MJ, Nodzenski M, Stevens RD, Ilkayeva O, Lowe LP, Metzger BE, Newgard CB, Scholtens DM, Lowe WL, for the HAPO Study Cooperative Research Group. 2017. Associations of maternal BMI and insulin resistance with the maternal metabolome and newborn outcomes. Diabetologia Springer Science and Business Media LLC 60:518–530. doi:10.1007/s00125-016-4182-2. PMC5300897 27981358

[26] Houttu N, Mokkala K, Laitinen K. 2018. Overweight and obesity status in pregnant women are related to intestinal microbiota and serum metabolic and inflammatory profiles. Clin Nutr 37:1955–1966. doi:10.1016/j.clnu.2017.12.013. 29338886

[27] Toivonen R, Emani R, Munukka E, Rintala A, Laiho A, Pietilä S, Pursiheimo J-P, Soidinsalo P, Linhala M, Eerola E, Huovinen P, Hänninen A. 2014. Fermentable fibres condition colon microbiota and promote diabetogenesis in NOD mice. Diabetologia 57:2183–2192. doi:10.1007/s00125-014-3325-6. 25031069

[28] Li J, Jia H, Cai X, Zhong H, Feng Q, Sunagawa S, Arumugam M, Kultima JR, Prifti E, Nielsen T, Juncker AS, Manichanh C, Chen B, Zhang W, Levenez F, Xu X, Xiao L, Liang S, Zhang D, Zhang Z, Chen W, Zhao H, Al-Aama JY, Eis S, Yang H, Hansen H, Nielsen HB, Brunak S, Kristiansen K, Guarner F, Pedersen O, Doré J, Ehrlich SD, Bork P, Wang J, de Vos WM, Tims S, Zoetendal EG, Kleerebezem M, MetaHIT Consortium. 2014. An integrated catalog of reference genes in the human gut microbiome. Nat Biotechnol 32:834–841. doi:10.1038/nbt.2942. 24997786

[29] Nielsen HB, Almeida M, Juncker AS, Rasmussen S, Li J, Sunagawa S, Plichta DR, Gautier L, Pedersen AG, Le Chatelier E, Pelletier E, Bonde I, Nielsen T, Manichanh C, Arumugam M, Batto JM, Quintanilha Dos Santos MB, Blom N, Borruel N, Burgdorf KS, Boumezbeur F, Casellas F, Doré J, Dworzynski P, Guarner F, Hansen T, Hildebrand F, Kaas RS, Kennedy S, Kristiansen K, Kultima JR, Léonard P, Levenez F, Lund O, Moumen B, Le Paslier D, Pons N, Pedersen O, Prifti E, Qin J, Raes J, Sørensen S, Tap J, Tims S, Ussery DW, Yamada T, Renault P, Sicheritz-Ponten T, Bork P, Wang J, MetaHIT Consortium, et al. 2014. Identification and assembly of genomes and genetic elements in complex metagenomic samples without using reference genomes. Nat Biotechnol 32:822–828. doi:10.1038/nbt.2939. 24997787

[30] Oksanen J, Blanchet FG, Friendly M, Kindt R, Legendre P, McGlinn D, Minchin PR, O’Hara RB, Simpson GL, Solymos P, Stevens HM, Wagner H. 2017. vegan: Community Ecology Package. https://CRAN.R-project.org/package=vegan.

[31] Love MI, Huber W, Anders S. 2014. Moderated estimation of fold change and dispersion for RNA-seq data with DESeq2. Genome Biol 15:550. doi:10.1186/s13059-014-0550-8. 25516281 PMC4302049

[32] Soininen P, Kangas AJ, Würtz P, Suna T, Ala-Korpela M. 2015. Quantitative serum nuclear magnetic resonance metabolomics in cardiovascular epidemiology and genetics. Circ Cardiovasc Genet 8:192–206. doi:10.1161/CIRCGENETICS.114.000216. 25691689

[33] Otvos JD, Shalaurova I, Wolak-Dinsmore J, Connelly MA, Mackey RH, Stein JH, Tracy RP. 2015. GlycA: a composite nuclear magnetic resonance biomarker of systemic inflammation. Clin Chem (Baltimore, Md) 61:714–723. doi:10.1373/clinchem.2014.232918. 25779987

[34] Katz A, Nambi SS, Mather K, Baron AD, Follmann DA, Sullivan G, Quon MJ. 2000. Quantitative insulin sensitivity check index: a simple, accurate method for assessing insulin sensitivity in humans. J Clin Endocrinol Metab 85:2402–2410. doi:10.1210/jcem.85.7.6661. 10902785

