# Supplementary material for: Distinct Diet-Microbiota-Metabolism Interactions in Overweight and Obese Pregnant Women: a Metagenomics Approach
Source: Microbiol Spectr. 2022 Mar 28;10(2):e00893-21. doi: 10.1128/spectrum.00893-21 (PMC9045358; doi:10.1128/spectrum.00893-21)
Supplement: SUPPLEMENTAL FILE 1 — Supplemental material. Download SPECTRUM00893-21_Supp_1_seq5.pdf, PDF file, 0.2 MB [file spectrum00893-21_supp_1_seq5.pdf]

Distinct diet-microbiota-metabolism interactions in overweight and obese pregnant women: a metagenomics approach

Mrunalini Lotankar, Kati Mokkala, Noora Houttu, Ella Koivuniemi, Nikolaj Sørensen, Henrik Bjørn Nielsen, Eveliina Munukka, Leo Lahti, Kirsi Laitinen

Supplementary material

Supplementary Table 1. Summary statistics for the sequencing reads

Reads	Average of reads (%)	95% Confidence Interval[a]
Mapped to host	0.48	0.28 - 0.69
Low quality reads	20.73	20.4 - 21.06
Unmapped reads	9.5	8.92 - 10.07
Mapped to gene catalog	69.29	68.61 - 69.97

[a] 95% Confidence Interval was calculated with CI() function from *Rmisc* package (36). Here, it represents the interval where 95% of data is located around the mean.

Supplementary Table 2. Most abundant species for the overweight and obese population

Bacterial species	Mean relative abundance (%)	
	**Overweight**	**Obese**
Clostridiales sp.	5.16	4.44
Bacteroides vulgatus	3.76	4.5
Bacteroides uniformis	2.89	2.35
Clostridia sp.	2.29	2.07
Bacteria sp.	2.04	1.3
Lachnospiraceae sp.	1.87	1.76
Firmicutes sp.	1.53	1.2
Alistipes finegoldii	1.47	1.7
Bacteroides dorei	1.45	1.34
Alistipes putredinis	1.41	1.53
Prevotella copri	1.3	1.68
Faecalibacterium prausnitzii	1.08	1.04
Faecalibacterium sp.	0.87	0.77

*Reads with an unknown taxonomy were reported as unmapped (if reads not mapped to the gene catalog) and orphan genes (if mapped to the gene catalog but not attributed to an MGS).

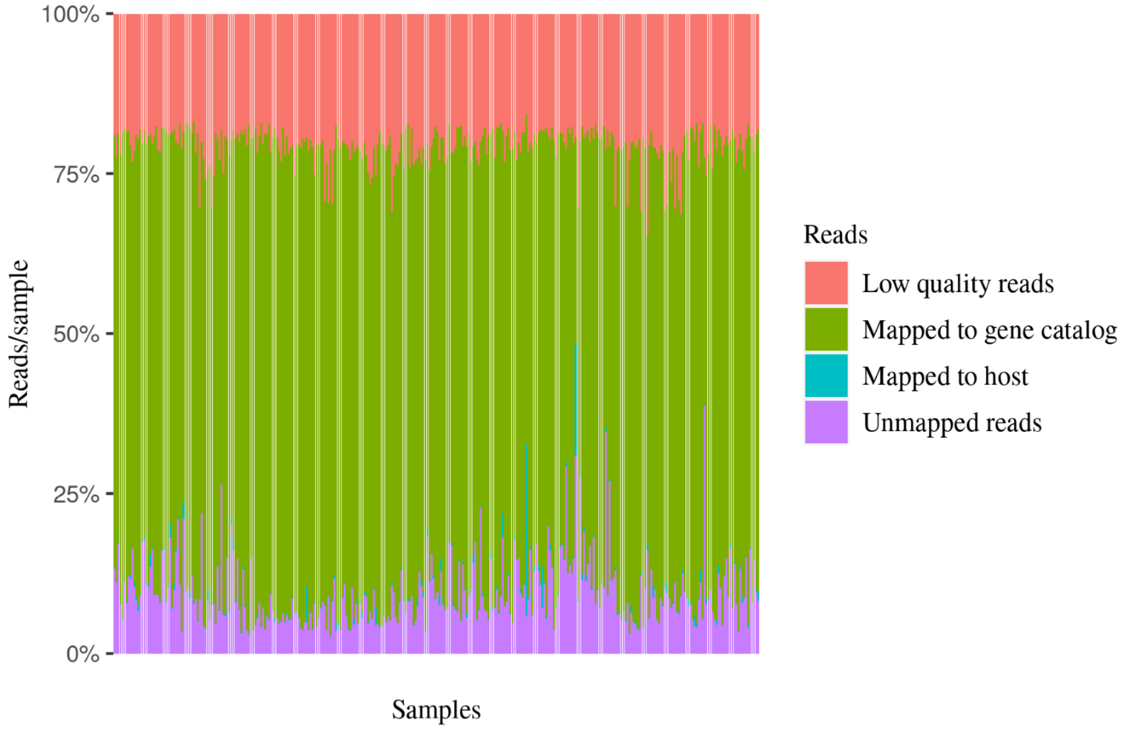

Supplementary figure 1. Sequence reads mapping information per sample. Number of reads (%) per sample: Figure shows percentages of reads that are of low quality, unmapped, mapped to host and mapped the gene catalog. Reads, which were of low quality, unmapped and mapped to the host genome, were discarded. Reads, which mapped to the gene catalog, were included for taxonomic annotation.

Reference for supplementary material

1. Hope RM. 2013. Rmisc: Ryan Miscellaneous. R Packag version 15.
